# Correlation analysis of prognostic and pathological features of patients with chronic sinusitis and nasal polyps following endoscopic surgery

**DOI:** 10.3892/etm.2013.1072

**Published:** 2013-04-23

**Authors:** JUN TANG, SUFU LIU, LIANGYUN ZHANG, WEIXIONG CHEN, SISI SHI, QINGQING YU, CHAO TANG, YUEJIAN WANG

**Affiliations:** 1 Department of Otolaryngology - Head and Neck Surgery, The First People’s Hospital of Foshan, Foshan, Guangdong 528000;; 2 Department of Otolaryngology - Head and Neck Surgery, Capital Medical University Affiliated Beijing Shijitan Hospital, Beijing 100038;; 3 Department of Pathology, The First People’s Hospital of Foshan, Foshan, Guangdong 528000, P.R. China

**Keywords:** sinusitis, nasal polyps, pathology, efficacy

## Abstract

The aim of this study was to evaluate the therapeutic value of pathological indicators to predict the efficacy of endoscopic sinus surgery (ESS) in patients with chronic rhinosinusitis (CRS) with nasal polyps. A total of 53 patients with CRS with nasal polyps, who had undergone endoscopic surgery at least one year before, were surveyed for their clinical symptoms. Surgical specimen biopsies were consulted and related pathological indicators were measured. The association between the main symptoms of CRS with nasal polyps following ESS and pathological indicators were statistically analyzed. The main symptoms of patients with CRS with nasal polyps following ESS were nasal congestion, thick nasal discharge, rhinorrhea or sneezing. Goblet cells are associated with the symptoms of sneezing and thick nasal discharge, pathological gland formation is associated with dizziness, and the degree of tissue edema is associated with post-nasal discharge (P<0.05). Pathological indicators aid the prediction of the efficacy of nasal ESS in patients with CRS with nasal polyps.

## Introduction

Chronic rhinosinusitis (CRS) with nasal polyps is a heterogeneous group of inflammatory diseases of the nasal and paranasal cavities (1). Histologically it manifests as an extremely edematous interstice covered with respiratory epithelium. Inflammatory cell infiltration occurs in the interstice, mainly eosinophil infiltration, accompanied by long tubular gland formation (1,2). Endoscopic sinus surgery (ESS) is a well-established strategy for the treatment of CRS, which does not respond to medical treatment. However, 5–10% of patients fail to respond to conventional ESS (3). Previous studies have shown that high-grade mucosal inflammation often results in a poor outcome (4). Cellular infiltration and local cytokine activity in the sinus mucosa collected at surgery may have important prognostic value for long-term outcome (5–7), Eosinophils, which contribute to mucosal injury by secretion of toxic granule proteins, including major basic protein and eosinophil cationic protein, play an important role in these procedures (8). However, the altered properties of the mucus in the nasal cavity decrease the function of the mucociliary clearance system. Goblet cells and subepithelial thickening are the histopathological parameters which correlate with the largest number of symptoms of allergic and non-allergic patients with CRS (9).

The aim of this study was to identify pathological indicators to predict the efficacy of ESS and to understand the pathological basis of refractory sinusitis.

## Subjects and methods

### Subjects

A total of 99 patients with CRS with nasal polyps underwent ESS by the same doctor at the Department of Otolaryngology, The First People’s Hospital of Foshan from January 2009 to December 2010. Of these, 53 patients who were able to return for a second visit and complete a survey questionnaire were selected, including 27 males and 26 females, ranging in age from 13 to 80 years (average, 36 years). All patients had nasal congestion, rhinorrhea, hyposmia and/or headaches and other symptoms persisting for >12 weeks. Anterior rhinoscopy or nasal endoscopy revealed middle meatus and/or olfactory cleft mucosal swelling, sticky purulent discharge and translucent neoplasm. Preoperative computed tomography (CT) revealed that the sinus mucosa had widespread or localized inflammatory lesions. Postoperative pathological biopsy indicated chronic inflammation of the mucosa and nasal polyp. This study was conducted in accordance with the Declaration of Helsinki and with approval from the Ethics Committee of the First Hospital of Foshan. Written informed consent was obtained from all participants.

### Symptomatology survey

The Sino-Nasal Outcome Test-20 (SNOT-20) was used as a reference to design a symptom survey questionnaire and scoring standard (10), including congestion, sneezing, rhinorrhea, cough, post-nasal discharge, thick nasal discharge, ear fullness, dizziness, ear pain, facial pain/pressure and olfaction. A total of 99 patients hospitalized in the Department of Otolaryngology, The First People’s Hospital of Foshan from January 2009 to December 2010 with CRS with nasal polyps who underwent ESS by the same physician were notified by telephone. Of the 99 patients, 53 patients returned to the hospital for follow-up investigation. The same investigator conducted the questionnaire survey and patients filled out the forms by themselves or under guidance.

### Pathological data survey

**Figure 1.**
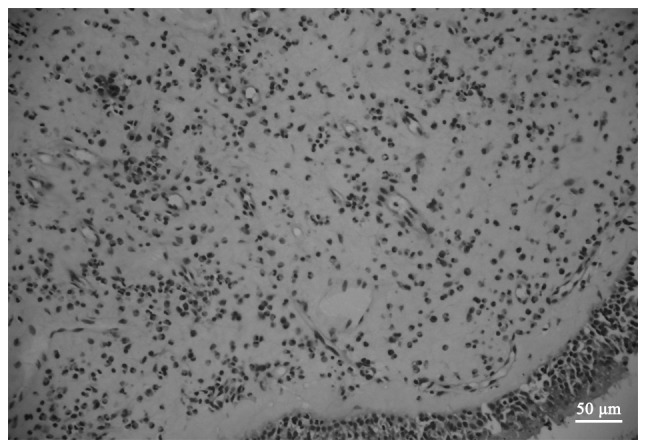
Nasal polyps biopsy, showing interstitial edema and infiltration of eosinophils (magnification, ×400).

**Figure 2.**
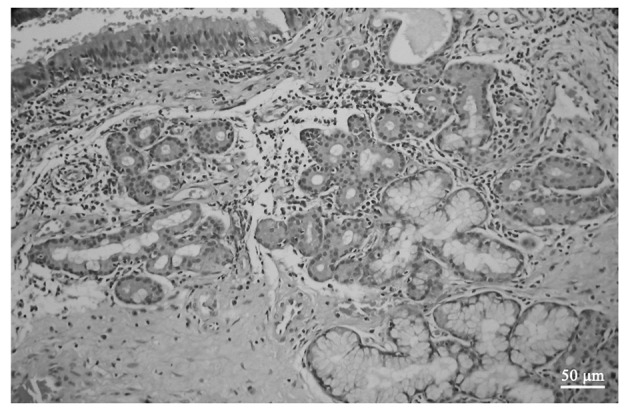
Pathological gland formation in nasal polyps (magnification, ×400).

The surgical specimen biopsies of 53 patients were retrospectively analyzed (Fig. 1) and graded. A semi-quantitative method was used to determine relevant pathological indicators, including eosinophils, lymphocytes, goblet cell infiltration density, edema of the submucosa and pathological mucosal gland density. Pathological glands refer to glands with lumen expansion, glands with cavities containing blue-staining mucinous secretions and glands with cystic dilatation (10,11), as shown in Fig. 2.

Specific grading criteria were as follows (9,12): i) Goblet cell infiltration density classification (high power field, magnification, ×400/0.144 mm^2^): 0, no goblet cells in the gland; 1, scattered goblet cells; 2, goblet cells diffusely distributed; and 3, flake-shaped goblet cells. ii) Lymphocytic infiltration density classification (high power field, magnification, ×400/0.144 mm^2^): 10 high power fields were counted and the average number of lymphocytes was calculated. The gradings were as follows: 0, 0–20; 1, 21–50; 2, 51–80; and 3, >80. iii) Eosinophil count (high power field, magnification, ×400/0.144 mm^2^): 10 high power fields were counted and the average number of eosinophils was calculated. The gradings were as follows: 0, 0–3; 1, 4–10; 2, 11–30; and 3, >30. iv) Mucosal pathological gland density (low magnification, ×100): 0, 0–3; 1, 4–10; 2, 11–30; and 3, >30. v) The degree of submucosal tissue edema (low magnification, ×100): 0, no edema; 1, mild edema; 2, moderate edema; and 3, significant edema.

### Statistical analysis

Correlation analysis between the main symptoms and the pathological indicators of patients with ESS was performed using Spearman’s rank correlation test. P<0.05 was considered to indicate a statistically significant difference.

## Results

### Common symptoms following ESS

The five most common symptoms of patients with CRS with nasal polyps following ESS are nasal congestion, runny purulent nasal discharge, sneezing, rhinorrhea and postnasal drip, successively.

### Correlation between symptoms and pathological indicators

The results of the statistical analysis of the correlation between pathological parameters and symptomatology are presented in Table I.

### Correlation between eosinophil infiltration density and symptoms

There was not determined to be a statistically significant correlation between eosinophil infiltration density and any of the symptoms (P>0.05).

### Correlation between lymphocyte infiltration density and symptoms

No statistically significant correlation was identified between the lymphocyte infiltration density and any of the symptoms (P>0.05).

### Correlation between goblet cell density and symptoms

There were statistically significant correlations between goblet cell density and sneezing and purulent nasal discharge (P=0.039 and P=0.036, respectively) with no correlation between goblet cell density with other symptoms.

### Correlation between the number of pathological glands and symptoms

The number of pathological glands and dizziness presented a statistically significant correlation (P=0.008) with no correlation between pathological glands and other symptoms.

### Correlation between degree of tissue edema and symptoms

The correlation between the degree of tissue edema and postnasal drip was revealed to be statistically significant (P=0.040) There was no correlation between degree of tissue edema and other symptoms.

## Discussion

The aim of this study was to identify pathological features associated with the outcome of ESS and to understand the pathological basis of refractory sinusitis. The study demonstrated that the most common nasal symptoms that distress patients following ESS are nasal congestion, runny purulent nasal discharge, rhinorrhea, postnasal drip and sneezing. Goblet cells and pathological glands are signification in the development of certain symptoms following ESS.

Lanza and Kennedy (13) suggested that since the diagnostic criteria of sinusitis are based on the local symptoms of the patient, indicators for determining the success or failure of chronic nasal-sinusitis surgery should also be based on the assessment of symptom relief. A number of questionnaires have been designed to evaluate the quality of life of patients and studies have shown that the health status of 88% of patients improved following ESS (14,15). The Medical Outcome Study Short Form-36 (SF-36) is a universal scale, which provides a good evaluation of a patient’s overall postoperative health situation. However, it also contains entries for emotional state and the correlation analysis for certain pathological conditions of the sinus mucosa is unrealistic. Therefore in the present study, we assessed local situations related to nasal symptoms using the SNOT-20 questionnaire. Since there was no entry concerning sense of smell among the questions, we added hyposmia to improve the assessment of nasal symptoms. In our study of 53 patients with CRS with nasal polyps 1–2 years after ESS, we identified that the most common nasal symptoms that distress patients following surgery are nasal congestion, runny purulent nasal discharge, sneezing, rhinorrhea and postnasal drip.

A previous study demonstrated that asthma or a history of allergic rhinitis, previous nose surgery and nasal polyps are adverse prognostic factors of ESS (4). In addition, CT grading of sinus disease, nasal partial cell infiltration and infiltration of cytokines, including an increase in eosinophils and interleukin (IL)-5 activity enhancement, indicate a poor prognosis (5). However, a study by Kountakis *et al* (15) demonstrated that preoperative and postoperative symptom scores (SNOT-20) of patients with CRS with nasal polyps are not related to the severity grading determined by CT, endoscopy and presence of asthma or allergy symptoms, since ∼25% of the imaging evidence indicated no clinical symptoms for sinusitis patients.

The pathology of CRS with nasal polyps is a chronic inflammatory disease. Endoscopic surgery removes irreversible lesions, opens blocked sinus ostia and creates conditions for the restoration of normal physiological function of the nasal cavity and sinus; however, in certain cases with poor outcomes, this is not successful. Local inflammation has a significant impact on surgical outcomes. Giger *et al* (16) identified that, in the middle turbinate mucosa, non-specific inflammatory cell (lymphocytes and plasma cells) infiltration density and nasal congestion (VSA measurement table and nasal resistance meter assessment) are closely related, which is important in determining the prognosis of ESS. The degree of infiltration of inflammatory cells in the middle turbinate mucosa is a good indicator of recurrence following surgery. Baudoin *et al* (17) considered that pathological parameters contribute to the prediction of the presence of certain chronic nasal-sinusitis symptoms.

In the present study, we conducted Spearman’s correlation analysis for postoperative symptoms and pathological indicators of patients with CRS with nasal polyps following nasal endoscopic surgery. We did not identify indicators related to all nasal symptoms. It is difficult to predict and assess nasal symptoms following surgery from only one pathological indicator. However, we identified pathological indicators of nasal polyp tissue that are associated with certain symptoms following ESS: i) The correlation of goblet cell density and sneezing and purulent nasal discharge is statistically significant (P>0.05) and goblet cells are pathological indicator that correlate the most with postoperative symptoms. Baudoin *et al* (9,17) identified that the density of goblet cells is associated with nasal congestion, rhinorrhea, itching, headache and coughing; therefore, goblet cells are considered most valuable for predicting postoperative symptoms. ii) The correlation between the number of pathological glands and dizziness was statistically significant (P=0.008). Airway epithelial goblet cells and mucosal lamina propria mucus glands produce mucus. The majority of scholars consider that in chronic nasal sinusitis, nasal congestion and an increase of viscous secretions are mainly due to the formation of new pathological glands, normal mucous gland hyperplasia and reactivity enhancement (10,11,18,19). However, the present study did not observe a statistically significant correlation between the number of pathological glands and an increase in nasal secretions. We consider that the presence of dizziness following ESS may be due to a large amount of viscous secretion that is difficult to discharge, leading to the obstruction of the sinus ostium. iii) The correlation between the degree of tissue edema and postnasal drip was statistically significant (P=0.040). iv) The correlation between the number of eosinophils and clinical symptoms was not statistically significant (P>0.05). Lacroix *et al* identified a significantly greater number of eosinophils in the polyps and lymphocytes of African patients while plasmocytes were abundant in polyps from Chinese and Caucasian individuals (20). In the present study, there was an increase in eosinophil number in the sinus mucous membrane of ∼50% of patients with CRS with nasal polyps. There are four main types of basic protein in eosinophil granule proteins, including ∼50% of major basic protein (MBP). MBP causes basophils and mast cells to release histamine, leading to a series of clinical symptoms, including sneezing, nasal itching and nasal congestion. However, in this group of patients, we did not identify a significant correlation between the degree of eosinophil infiltration and clinical symptoms. v) The correlation between lymphocytic infiltration density and all clinical symptoms was not statistically significant (P>0.05).

In the current study, we identified that overall improvement of patient symptoms with CRS with nasal polyps does not rely solely on the pathological indicators; however, some pathological indicators have a certain value in forecasting certain nasal symptoms. Previous outcome studies have shown that patients with a poor outcome and high-grade inflammatory load may show improved results with more radical surgery (21–23). More studies are required to determine whether patients with a greater number of goblet cells and pathological glands require more radical surgery.

## Figures and Tables

**Table I.** P-values for Spearman’s rank correlation analysis of subjective symptoms and pathological parameters of patients following endoscopic sinus surgery.

Symptoms	Eosinophils		Lymphocytes		Goblet cells		Pathological glands		Tissue edema	
	P-value	Correlation coefficient	P-value	Correlation coefficient	P-value	Correlation coefficient	P-value	Correlation coefficient	P-value	Correlation coefficient
Nasal congestion	0.349	0.131	0.317	0.140	0.070	0.251	0.625	0.069	0.128	0.212
Sneezing	0.087	0.237	0.959	0.007	0.039^a^	0.284	0.547	0.085	0.230	0.168
Rhinorrhea	0.499	0.095	0.602	0.073	0.815	0.033	0.476	0.100	0.542	0.086
Coughing	0.757	0.044	0.143	0.204	0.799	0.036	0.839	0.029	0.558	0.082
Postnasal drip	0.124	0.214	0.421	0.113	0.906	0.017	0.102	0.227	0.040^a^	0.283
Purulent nasal discharge	0.129	0.211	0.692	0.056	0.036^a^	0.289	0.773	0.041	0.398	0.118
Ear fullness	0.465	0.102	0.957	0.008	0.706	0.053	0.361	0.128	0.431	0.110
Dizziness	0.344	0.133	0.360	0.128	0.227	0.169	0.008^a^	0.359	0.088	0.237
Ear pain	0.796	0.036	0.652	0.063	0.976	0.004	0.847	0.027	0.918	0.015
Facial pain	0.824	0.031	0.890	0.020	0.694	0.055	0.728	0.049	0.164	0.194
Hyposmia	0.931	0.012	0.280	0.151	0.267	0.155	0.668	0.060	0.419	0.113

Correlation analysis of the clinical symptoms and pathological parameters;

a P<0.05 was considered to indicate a statistically significant correlation.
